# Impact of an Order Set on Communication and Length of Stay After Pediatric Spinal Fusion

**DOI:** 10.7759/cureus.115662

**Published:** 2026-09-02

**Authors:** Jennifer W Glusman, Mark Kiley, David Roberts, Michelle Rice, Kevin Baker, Mahbubul Hasan, Anne Doran

**Affiliations:** 1 Hospital Medicine, Advocate Children’s Hospital, Park Ridge, USA; 2 Pediatric Orthopedics, Endeavor Health, Park Ridge, USA; 3 Nursing, Advocate Children’s Hospital, Park Ridge, USA; 4 Pediatrics, Advocate Children’s Hospital, Park Ridge, USA; 5 Graduate Medical Education, Advocate Aurora Healthcare, Park Ridge, USA

**Keywords:** interdisciplinary communication, pediatric spinal fusion, postoperative care, quality improvement projects, standardized order set

## Abstract

Background

This study aimed to evaluate the impact of a standardized postoperative order set on interdisciplinary communication and hospital length of stay (LOS) among pediatric patients undergoing posterior spinal fusion (PSF) for idiopathic scoliosis.

Methodology

LOS was compared among patients younger than 18 years using Epic SlicerDicer data from the pre-implementation period (2021 through January 17, 2024; n = 58) and the post-implementation period (January 18 through December 2024; n = 14). Pediatric residents were surveyed regarding the frequency of interdisciplinary communication before and after implementation. Statistical analyses used Fisher’s exact test and an independent-samples t-test.

Results

Mean LOS decreased from 3.91 days pre-implementation to 3.71 days post-implementation, though not statistically significant (p = 0.60). Survey responses demonstrated significant reductions in paging frequency for pain management (p = 0.043), drain output (p = 0.004), and pediatric-to-orthopedic communication regarding blood pressure concerns (p = 0.010). Overall interdisciplinary communication between pediatrics, orthopedics, and nursing improved.

Conclusions

Implementation of a standardized postoperative order set for pediatric patients undergoing PSF for idiopathic scoliosis significantly reduced unnecessary paging related to pain management, blood pressure monitoring, and drain output, while enhancing interdisciplinary communication. A trend toward shorter LOS was observed. These findings align with prior evidence that structured, diagnosis-specific order sets improve care coordination, efficiency, and patient outcomes in pediatric inpatient settings.

## Introduction

Posterior spinal fusion (PSF) is the standard of care for patients with severe adolescent idiopathic scoliosis (AIS), providing reliable deformity correction and restoration of coronal and sagittal alignment [1]. Advances in surgical techniques, pedicle screw instrumentation, and perioperative management have contributed to significant improvements in surgical safety and long-term outcomes [2]. However, postoperative care for pediatric spinal fusion patients remains complex, requiring close coordination among orthopedic surgeons, pediatric hospitalists, nursing staff, and ancillary services to ensure effective pain control, early mobilization, and patient safety. Multidisciplinary care pathways have been shown to reduce complications, shorten hospital length of stay (LOS), and improve patient and family satisfaction in this population [3].

Despite these advances, PSF is associated with substantial postoperative pain and a broad range of potential complications, including blood loss, neurologic deficits, respiratory complications, gastrointestinal dysfunction, infection, pseudarthrosis, and instrumentation failure [4,5]. Pain management is a central focus of postoperative care, as patients undergoing PSF typically require higher analgesic doses than most other pediatric surgical populations. Contemporary management strategies emphasize multimodal pain control, incorporating opioid and non‑opioid medications alongside nonpharmacologic interventions such as early mobilization and supportive care, which have been shown to improve recovery and reduce LOS [3,6]. Additional postoperative considerations include respiratory support to prevent atelectasis and pneumonia, infection prevention through standardized wound care and antibiotic protocols, proactive bowel management, and vigilant nursing assessments to detect early complications [3,4].

Effective interdisciplinary communication is critical to coordinating these diverse aspects of care, as nurses frequently serve as central communicators between pediatric hospitalists, orthopedic surgeons, and allied health professionals. Communication failures can lead to delays in intervention, increased paging burden, and decreased efficiency in care delivery [7]. Structured communication frameworks and standardized care tools, such as order sets, have been shown to improve clarity, reduce variability, and support shared understanding among care team members [8,9]. The widespread adoption of electronic medical records has facilitated the development of standardized, diagnosis‑specific order sets that function as clinical pathways, clearly outlining postoperative expectations and treatment milestones [10,11]. Such tools have been associated with improved interprofessional communication, reduced medical errors, and shorter hospital stays in pediatric inpatient settings [10,11].

At our institution, postoperative management following PSF varied in several areas based on individual surgeon preferences, including urinary catheter management, activity progression, pain management strategies, and timing of postoperative imaging. In addition, important aspects of care such as bracing instructions, blood pressure parameters, showering restrictions, positioning, and activity limitations frequently required clarification among nursing staff, pediatric providers, and orthopedic surgeons. This variability created communication inefficiencies and increased paging burden during the postoperative period.

In 2024, our institution implemented a comprehensive standardized postoperative order set for pediatric patients undergoing PSF for idiopathic scoliosis following multidisciplinary collaboration among orthopedics, anesthesia, pediatrics, and nursing. This study aims to evaluate the impact of this order set on interdisciplinary communication between pediatric hospitalists, orthopedic surgeons, and nursing staff, as well as on the efficiency of care delivery as measured by hospital LOS. We hypothesized that implementation of a standardized postoperative order set would improve interdisciplinary communication among care team members and reduce hospital LOS for pediatric patients undergoing PSF.

## Materials and methods

Study design and patient population

This was a retrospective pre- and post-intervention study evaluating the impact of the order set on LOS and provider communication between nurses and pediatric residents within pediatric hospital medicine and between pediatric residents and orthopedic surgery clinicians. The primary outcome was interdisciplinary communication as measured by resident survey responses. The secondary outcome was hospital LOS. Patients included were admitted for surgery for AIS, treated with PSF with instrumentation at thoracic or lumbar levels, under 18 years of age, and admitted to the pediatric intensive care unit or general pediatric service of our children’s hospital.

Patients were excluded if they underwent anterior spinal fusion, multiple surgeries during the same admission, used halo-gravity traction, or had other diagnoses such as neuromuscular disorders, congenital scoliosis, autism, genetic syndromes, congenital heart disease, or bleeding disorders.

LOS and other demographic data were measured using Epic’s SlicerDicer tool, comparing patients in the pre-intervention period (2021 through January 17, 2024) with those admitted during the post-intervention period (January 18 through December 2024). This study was reviewed by the Advocate Children’s Hospital, Park Ridge Institutional Review Board and was determined to be exempt from review. The requirement for informed consent was waived.

Provider communication survey

To assess the impact of the order set on interdisciplinary communication, pediatric residents who had cared for postoperative spinal fusion patients were surveyed before and after implementation. The survey was distributed electronically through REDCap and included questions regarding the frequency of communication between residents and nursing staff and between residents and orthopedic surgeons. Communication was defined as any page, phone call, or in-person interaction. Topics assessed included pain management, ambulation, blood pressure parameters, JP drain output, and bracing. The survey was developed for this quality improvement initiative and was not formally validated or pilot tested. Survey responses were obtained from 14 residents before implementation and 11 residents after implementation.

Order set development and implementation

A standardized postoperative spinal fusion order set was developed collaboratively by the departments of Orthopedic Surgery, Pediatric Hospitalist Medicine, and Pediatric Anesthesia at our children’s hospital (Table 1). To address variability in postoperative management practices and communication, surgeon-specific preferences were incorporated into the order set, which was placed by the orthopedic surgery team before or upon PICU admission. The order set was designed to streamline and standardize postoperative care for pediatric patients undergoing PSF. Orders were structured to dynamically adjust based on the patient’s postoperative day, with components added or discontinued accordingly.

**Table 1:** Standardized postoperative order set for pediatric spine deformity surgery (Epic Build). POD = postoperative day; PCA = patient-controlled analgesia; CBC = complete blood count; CMP = comprehensive metabolic panel; BMP = basic metabolic panel; PT = prothrombin time; PTT = partial thromboplastin time; CXR = chest X-ray; XR = X-ray; MRSA = methicillin-resistant *Staphylococcus aureus*; GI = gastrointestinal; PT/OT = physical therapy/occupational therapy; Hgb = hemoglobin; SBP = systolic blood pressure; MAP = mean arterial pressure

Category	POD 0	POD 1	POD 2	POD 3+
Pain management	Start PCA per pain service	Continue PCA and pain service management	Continue PCA or transition per pain service	Continue pain service plan
Labs/Imaging	STAT: CBC w/diff, CMP or BMP, magnesium, PT, PTT, D-dimer, fibrinogen. Optional: Imaging PRN: CXR, scoliosis XR cervical/thoracic/lumbar spine XR, CT/MRI spine	AM: CBC w/diff, CMP or BMP, magnesium	–	Repeat only if clinically indicated
Antibiotics	Cefazolin 30 mg/kg IV q8h ×2 doses. Alternatives: cefazolin + vancomycin (if MRSA), vancomycin alone (if severe allergy), or no antibiotics	Complete 2nd doses if not already given	–	–
GI/Bowel regimen	Famotidine 0.5 mg/kg IV q12h ×2 doses (max 20 mg), then PO. Senna PO BID. Docusate PO BID	Famotidine PO 20 mg q12h. Senna + Colace BID	Continue Famotidine PO 20 mg q12h. Senna + Colace BID. Add Miralax 17 g PO BID	Famotidine PO 20 mg q12h. Continue Senna, Colace, Miralax until return of bowel function. Optional: Bisacodyl suppository PR daily PRN (5 mg age 2–11; 10 mg age ≥12)
Activity/PT-OT	Activity as tolerated. Log roll. HOB 30°. Supine permitted. PT/OT consults same day. Optional: Shower allowed with OnQ ball	Begin ambulation as tolerated	Increase ambulation	Continue PT/OT and ambulation
Urinary catheter	Maintain if indicated for urine output monitoring. Discontinue per protocol. Foley timing options: AM POD1, POD2, bedtime, etc.	Remove Foley if ambulatory. Optional: Intermittent cath PRN if unable to void (>350 mL residual)	Optional: Reassess removal if still in place. Bladder scan PRN	–
Drain care	Record drain output q8h; notify if >250 mL/8 h. Reinforce dressing PRN. Barrier precautions if wound drainage not contained. Optional: ice to incision q2h	Continue monitoring and dressing care. Optional: May disconnect suction to ambulate	Continue monitoring. Notify if >250 mL/shift	Optional: Remove drains when criteria met
Thoracic bracing	No brace. Alternatives: TLSO brace per surgeon order (strict vs modified). Soft collar, or hard collar. May allow removal for hygiene, shower, bathroom	Continue brace use when ambulating	Continue brace per protocol	Continue or discontinue per surgical team
Antiemetics (PRN)	Ondansetron 0.1 mg/kg IV q8h (max 4 mg). Optional: Metoclopramide 0.1 mg/kg IV q6h PRN (max 10 mg)	Ondansetron or Metoclopramide PRN	PRN antiemetics	PRN antiemetics
Transfusion guidelines	Transfuse for Hgb <7 g/dL. Hold anticoagulants if platelets <100,000 or chest tube output >300 mL/6 h. Alternative: hold anticoagulants if platelets <50,000 with chest tube output >300 mL/6 h	–	–	–
Blood pressure management	Maintain SBP ≥90 mmHg and MAP ≥85 for 24 h postoperatively. Notify physician if not met. Optional: Extend duration to 48 h or until discontinued	–	–	–
Nursing communications	I/O monitoring. Neuro checks q15 min ×1 h → q30 min ×1 h → q1 h ×24 h. Encourage incentive spirometry. q1h while awake. Optional: SCDs. Notify physician if bladder management needed	Continue neuro checks and incentive spirometry	Continue incentive spirometry	Continue neuro checks and vital signs per unit routine; incentive spirometry

The order set included comprehensive management instructions for multimodal pain management, bowel regimen, urinary catheter care, early mobilization and ambulation, physical and occupational therapy, drain management, thoracic bracing if indicated by the surgeon, laboratory and imaging studies, antibiotic use, blood pressure parameters, and transfusion thresholds (Table 1). Surgeon-specific therapy preferences for three orthopedic surgeons were incorporated to ensure universal adoption. The order set was implemented on January 18, 2024, at both children’s hospital campuses, although only studied at the Park Ridge location.

Statistical analysis

Statistical analyses were performed to compare outcomes before and after implementation of the standardized postoperative order set. Continuous variables, including patient age and hospital LOS, were summarized using means and standard deviations. Welch’s two-sample t-test was used to compare continuous variables because of the unequal sample sizes between the pre- and post-implementation groups and the potential for unequal variances. Categorical variables were summarized using counts and percentages. Pearson’s chi-squared test was used to compare LOS distributions between groups. Fisher’s exact test was used for survey variables because of the small sample size and low expected cell counts within several response categories. All statistical tests were two-sided, and a p-value <0.05 was considered statistically significant.

## Results

The average LOS was evaluated across four calendar years (2021-2024) and two different pediatric orthopedic surgeons (Table 2) in a single center. A total of 72 patients were included in the analysis, with 58 admissions occurring before and 14 after implementation of the order set. Baseline groups before/after implementation were similar: the mean age of patients between the groups was comparable (14 ± 2 years before vs. 15 ± 2 years after; p = 0.30) (Table 3), with a Welch t‑test yielding a t of −1.05, indicating no statistically significant difference in mean age between the pre‑ and post‑implementation groups.

**Table 2:** LOS before and after order set implementation. LOS before and after order set implementation. Values are presented as N (%). P‑values were calculated using Pearson’s chi‑squared test. A p‑value <0.05 was considered statistically significant. LOS = length of stay

Characteristic	Before (N = 58)	After (N = 14)	P-value
LOS (days)	—	—	0.91
2	10 (17.2%)	2 (14.3%)	—
3	18 (31.0%)	4 (28.6%)	—
4	12 (20.7%)	5 (35.7%)	—
5	13 (22.4%)	2 (14.3%)	—
6	3 (5.2%)	1 (7.1%)	—
8	1 (1.7%)	0 (0%)	—
14	1 (1.7%)	0 (0%)	

**Table 3:** Patient demographics comparison: age and LOS before versus after order set implementation. Patient demographic characteristics and length of stay before and after order set implementation. Values are presented as mean (SD). Welch’s two‑sample t‑test was used to compare continuous variables. A p‑value <0.05 was considered statistically significant. ^1^: Mean (SD); n/N (%); ^2^: Welch’s two‑sample t‑test; Pearson’s chi-squared test. LOS = length of stay

Statistical differences before and after order set implementation	Before, n = 58^1^	After, n = 14^1^	t-value	P-value^2^
Age (years)	14 (2)	15 (2)	−1.05	0.3
LOS (days)	3.91 (1.87)	3.71 (1.14)	0.52	0.6

The mean LOS was 3.9 ± 1.9 days before implementation and 3.7 ± 1.1 days following order set implementation, with a Welch t‑test yielding a t of 0.52, showing a modest reduction in LOS following implementation of the order set; however, this difference was not statistically significant (p = 0.60).

Measures of communication showed significant improvements. Comparison of nursing and orthopedic resident communication before (n = 14) and after (n = 11) the order set implementation revealed statistically significant changes in several areas, based on Fisher’s exact tests. Notably, communication between nursing and pediatric residents regarding drain management (p = 0.004) and patient pain (p = 0.043), as well as communication between pediatric residents and orthopedic surgery regarding blood pressure management (p = 0.010), showed a significant decrease (Tables 4, 5). Trends toward significance were also observed for communication regarding bracing between nurses and pediatric residents (p = 0.056) and between pediatric residents and orthopedic surgery (p = 0.087), though these did not reach conventional thresholds for statistical significance (Tables 4, 5). These findings suggest that the order set positively influenced resident and nursing communication behavior, particularly in areas related to bracing, drain management, blood pressure monitoring, and pain assessment.

**Table 4:** Nursing-to-pediatric resident communication before versus after order set implementation. Nursing-to-pediatric resident communication before and after order set implementation. Values are presented as N (%). P‑values were calculated using Fisher’s exact test. *: Indicates a statistically significant p‑value of <0.05.

Survey item	Before (n = 14)						After (n = 11)						Mean (before)	Mean (after)	P-value
Number of points of contact/Day	0	1	2	3	4	5	0	1	2	3	4	5			
General daily	0 (0%)	3 (21%)	3 (21%)	6 (43%)	0 (0%)	2 (14%)	0 (0%)	6 (55%)	3 (27%)	2 (18%)	0 (0%)	0 (0%)	2.64	1.64	0.2
Pain	0 (0%)	0 (0%)	3 (21%)	4 (29%)	3 (21%)	4 (29%)	1 (9%)	3 (27%)	5 (45%)	1 (9%)	1 (9%)	0 (0%)	3.57	1.82	0.043*
Ambulation	1 (7%)	5 (36%)	3 (21%)	3 (21%)	0 (0%)	2 (14%)	3 (27%)	4 (36%)	3 (27%)	1 (9%)	0 (0%)	0 (0%)	2.14	1.18	0.6
Number of points of contact/admission	Never		Every other admission		Every admission		Never		Every other admission		Every admission		—	—	—
Bracing	5 (36%)		7 (50%)		2 (14%)		9 (82%)		2 (18%)		0 (0%)		0.79	0.2	0.056
Drain	0 (0%)		10 (71%)		4 (29%)		5 (45%)		6 (55%)		0 (0%)		1.29	0.6	0.004*
Blood pressure	2 (14%)		8 (57%)		4 (29%)		6 (55%)		3 (27%)		2 (18%)		1.14	0.7	0.14

**Table 5:** Pediatric resident-to-orthopedics communication before versus after order set implementation. Pediatric resident-to-orthopedic surgeon communication before and after order set implementation. Values are presented as N (%). P‑values were calculated using Fisher’s exact test. *: Indicates a statistically significant p‑value of <0.05.

Survey item	Before (n = 14)						After (n = 11)						Mean (before)	Mean (after)	P-value
Number of points of contact/day	0	1	2	3	4	5	0	1	2	3	4	5			
General daily	2 (14%)	7 (50%)	3 (21%)	1 (7%)	0 (0%)	1 (7%)	1 (9%)	8 (73%)	0 (0%)	1 (18%)	0 (0%)	0 (0%)	1.5	1.27	0.6
Pain	5 (36%)	2 (14%)	4 (29%)	2 (14%)	0 (0%)	1 (7%)	4 (36%)	5 (45%)	1 (18%)	0 (0%)	0 (0%)	0 (0%)	1.5	0.82	0.4
Number of points of contact/admission	Never		Every other admission		Every admission		Never		Every other admission		Every admission		—	—	—
Ambulation	4 (29%)		7 (50%)		3 (21%)		6 (55%)		5 (45%)		0 (0%)		0.93	0.5	0.3
Bracing	3 (21%)		9 (64%)		2 (14%)		7 (64%)		4 (36%)		0 (0%)		0.93	0.4	0.087*
Drain	2 (14%)		9 (64%)		3 (21%)		5 (45%)		6 (55%)		0 (0%)		1.07	0.6	0.2
Blood pressure	6 (43%)		35 (36%)		3 (21%)		11 (100%)		0 (0%)		0 (0%)		0.79	0.0	0.01*

## Discussion

This quality improvement initiative demonstrates that implementation of a standardized postoperative order set for pediatric spinal fusion patients enhanced interdisciplinary coordination by reducing unnecessary communication related to ambulation, bracing, pain management, drain care, and blood pressure parameters. Many of these improvements reached statistical significance despite the modest sample size.

Notably, the communication domains demonstrating significant improvement, including pain management, drain management, and blood pressure monitoring, corresponded directly to topics addressed within the standardized order set. This finding supports the plausibility that the intervention contributed to the observed communication changes, although causality cannot be definitively established.

Postoperative management of spinal fusion patients is inherently complex, requiring coordination among surgeons, pediatric hospitalists, nursing staff, and ancillary services. Prior variability in surgeon preferences often resulted in inconsistent care and frequent communication. Standardizing care expectations reduced ambiguity and enabled frontline providers to deliver care more consistently, resulting in a reduced communication burden for common postoperative issues.

Existing literature supports the role of diagnosis-specific order sets in improving care coordination, reducing cognitive burden, and streamlining communication. Systematic reviews have demonstrated that clinical pathways are associated with reduced hospital LOS and improved efficiency without adverse effects on patient outcomes [12]. Similar benefits have been observed following the implementation of electronic order sets in pediatric inpatient care [11].

In addition to improved communication, we observed a modest reduction in LOS following order set implementation; however, this difference was not statistically significant. While the direction of the change is consistent with prior studies demonstrating reductions in LOS following implementation of standardized clinical pathways and recovery protocols, our findings do not provide evidence that the order set independently reduced LOS in this cohort [12,13]. Larger studies are needed to determine whether implementation of a standardized postoperative order set results in clinically meaningful improvements in LOS.

Strong interdisciplinary collaboration throughout development and implementation helped ensure that the order set reflected the needs of frontline providers and integrated into existing clinical workflows. Similar findings have been reported in enhanced recovery and rapid recovery pathways for pediatric spinal fusion, which have been associated with decreased LOS, improved pain control, and greater standardization of postoperative care [3,6]. Our findings extend this work by demonstrating that standardizing postoperative orders can also reduce the need for clarification across disciplines, suggesting a complementary role for communication-focused interventions within broader multimodal recovery strategies.

Several limitations should be considered. This single-center study included a small post-intervention cohort, limiting statistical power to detect differences in LOS. The post-intervention period was intentionally limited to avoid overlap with a subsequent institutional initiative targeting postoperative spinal fusion care. Including patients exposed to that initiative could have confounded evaluation of the order set. In addition, as a retrospective pre-post study, unmeasured factors such as changes in perioperative management, staffing, or other clinical practice patterns may have influenced communication and LOS independent of the intervention. The communication survey was developed specifically for this quality improvement project and was not formally validated. Survey participation was voluntary and may have introduced response and recall bias. Detailed clinical and operative characteristics, including sex, curve severity, fusion levels, operative time, estimated blood loss, transfusion requirements, and comorbidities, were not available for analysis. Consequently, comparability between cohorts could only be assessed using available demographic variables, and residual confounding may have influenced the observed outcomes. Additional clinical outcomes, including patient satisfaction, complications, readmissions, opioid utilization, pain control, return of bowel function, and mobilization milestones, were not evaluated and warrant further study. Future investigations incorporating these measures may better define the clinical impact of standardized postoperative order sets.

What distinguishes this work is its focused evaluation of a specialized pediatric surgical population combined with direct assessment of real-time communication behaviors among frontline providers. Few studies have examined the operational impact of order sets on interdisciplinary communication at the level of daily nursing and resident workflows. By integrating objective and experiential data, this study offers insight into how structured order sets influence both care delivery and team function in a tertiary pediatric setting [14,15].

## Conclusions

Implementation of a standardized postoperative order set for pediatric patients undergoing PSF for idiopathic scoliosis was associated with improved interdisciplinary communication. Although LOS was numerically lower following implementation, no statistically significant difference was observed. By reducing uncertainty around common postoperative management decisions, the order set reduced clarification-related communication and supported more efficient coordination among pediatric residents, orthopedic surgeons, and nursing staff. Beyond measurable outcomes, this intervention highlights the importance of designing care tools that align with frontline clinical workflows. Embedding clear expectations for postoperative care within the electronic medical record helped promote consistency, enhance teamwork, and reinforce shared responsibility across disciplines. While larger studies are needed to better evaluate effects on LOS and downstream outcomes, standardized, diagnosis-specific order sets represent a practical and scalable strategy to improve communication, efficiency, and safety in complex pediatric surgical populations.
